# Evaluating the agreement of continuous glucose monitoring system with venous methods for glycemic index determination

**DOI:** 10.3389/fnut.2026.1842475

**Published:** 2026-05-28

**Authors:** Lihua Li, Qiaosheng Hu, Menglan Li, Yan Zhao, Rui Zhang, Yan Liu, Xin Tong, Haiying Zhu, Mengya Zhang, Qing Zhao, Xianghua Ma

**Affiliations:** 1 Lianshui People’s Hospital Affiliated to Kangda College of Nanjing Medical University, Huai’an, Jiangsu, China; 2 Jiangsu Province People’s Hospital, The First Affiliated Hospital with Nanjing Medical University, Nanjing, Jiangsu, China

**Keywords:** continuous glucose monitoring system, glycemic index, incremental area under the curve, Lin’s concordance correlation coefficient, Parkes error grid

## Abstract

**Background:**

Continuous glucose monitoring systems (CGMS) are widely used in diabetes care, but their reliability for standardized food glycemic index (GI) measurement remains unclear. Most previous studies used capillary blood rather than venous glucose as the reference.

**Objective:**

This study aimed to verify the consistency between the CGMS and standardized venous sampling methods for measuring postprandial incremental area under the curve (IAUC) and GI values.

**Methods:**

This study was conducted in accordance with the Chinese GI testing standard WS/T 652–2019. Three measurement methods were compared: venous sampling (Method A), CGMS with all data points adopted (Method B), and CGMS data aligned with the standard venous time points (Method C). One-way analysis of variance (ANOVA) and Lin’s concordance correlation coefficient (CCC) were used for statistical analysis. The Parkes Error Grid analysis was employed to ascertain the clinical acceptability of glucose discrepancies within low, medium, and high concentration strata.

**Results:**

The 12 enrolled participants (six males and six females) had a mean age of 29.50 ± 10.12 years, mean BMI of 21.5 ± 1.7 kg/m^2^, mean HbA1c of 5.07 ± 0.21%, and all lipid, glycemic, hepatic and renal function indicators were within normal reference ranges. For 120-min IAUC, the mean values were 186.93 ± 87.29 (Method A), 166.00 ± 76.46 (Method B) and 164.24 ± 77.48 (Method C). ANOVA showed no significant inter-method difference (*F* = 1.178, *p* = 0.311). CCC analysis with Method A as the reference revealed good agreement: CCC = 0.8818 (95% CI: 0.8068–0.9289) for A vs. B and CCC = 0.8727 (95% CI: 0.7921–0.9234) for A vs. C, with a high Pearson’s *ρ* (>0.91) and bias correction factors (Cb, >0.95 fot both). For GI values (n = 12 for each method), the mean values were 55.07 ± 15.57 (Method A), 54.28 ± 12.24 (Method B) and 53.68 ± 15.04 (Method C). ANOVA showed no significant inter-method difference (*F* = 0.028, *p* = 0.972), and CCC analysis indicated moderate agreement (CCC ≈ 0.79) with minimal systematic bias (Cb > 0.97).

**Conclusion:**

CGMS methods demonstrate good-to-moderate concordance with standardized venous testing for IAUC and GI determination. CGMS may represent a feasible, non-invasive alternative for food GI evaluation in nutritional and diabetes-related research among healthy individuals.

## Introduction

The 2025 Global Diabetes Map (1) reported that approximately 589 million adults worldwide are affected by diabetes mellitus (DM), with more than 95% of these patients diagnosed with type 2 diabetes mellitus (T2DM), accounting for 148 million cases in China. Nutritional therapy remains a cornerstone of the “five pillars” of diabetes management (2). Adoption of a low glycemic index (LGI) diet can mitigate postprandial hyperglycemia and reactive hyperinsulinemia, thereby contributing to the prevention and management of hyperglycemia-related comorbidities, including T2DM, coronary artery disease, obesity, hypertension, dyslipidemia, and certain malignant neoplasms (3–7).

The glycemic index (GI) quantifies the relative glycemic response to a specific carbohydrate load. It is defined as the ratio of the incremental area under the blood glucose response curve (IAUC) following the ingestion of a test food containing 50 grams of carbohydrates to that following the consumption of 50 grams of anhydrous glucose in healthy subjects. Blood glucose concentrations are systematically measured at baseline (0 min) and at postprandial intervals of 15, 30, 45, 60, 90, and 120 min. This methodology facilitates the assessment of various foods’ impact on postprandial glycemia (8).

Continuous glucose monitoring system (CGMS) facilitates personalized, real-time assessment of glycemic profiles by analyzing glucose concentrations in interstitial fluid. CGMS are considered the standard of care for patients with type 1 diabetes mellitus (T1DM), and Ramzi’s research (9) has explored the potential benefits of CGMS application in patients with T2DM. CGMS provided more comprehensive data on circadian glucose fluctuations in daily life, facilitating the identification of abnormal glucose metabolism in healthy populations and supports personalized risk assessment and intervention decisions. Its practical application value has been acknowledged in relevant discussions (10). In contrast to traditional measurement techniques, which possess significant limitations in meeting the individualized and intelligent requirements of precision medicine, CGMS can provide 24-h, continuous glucose monitoring, with the advantages of minimal invasiveness or even non-invasiveness (11–13). This technology reduced the burden of frequent fingerstick blood sampling and enhances patient adherence and satisfaction (14, 15).

Prior to the project’s commencement, the research team conducted statistical analyses comparing selected CGMS glucose values with venous sampling plasma glucose values. The study involved 26 volunteers, generating a total of 234 paired measurements of dynamic glucose and venous glucose values at different time points. Statistical analysis of this data revealed a high degree of concordance between the two glucose measurement methods, with no statistically significant difference observed between the groups (*F* = 0.753, *p* = 0.386). Furthermore, the Lin’s Concordance Correlation Coefficient (CCC) and its 95% confidence interval were 0.8877 (0.8579–0.9116), the Pearson *ρ* coefficient was 0.8956, and the bias correction factor Cb was 0.9912.

Current research on the utilization of CGMS in healthy populations is relatively limited. This research will employ CGMS in healthy, normal-weight participants to assess postprandial glycemic responses, thereby exploring the utility of CGMS for evaluating the GI of foods. This study will compare GI values of the same test food obtained by conventional venous sampling with those derived from CGMS and evaluate the feasibility of integrating CGMS into clinical and nutritional GI assessment protocols.

## Methods

### Study designs and participants

This clinical trial was conducted at Lianshui People’s Hospital Affiliated to Kangda College of Nanjing Medical University from Dec 2025 to Feb 2026.

This study was performed in accordance with the Chinese national industry standard WS/T 652–2019 entitled “Method for Determining the Glycemic Index of Food” (16). The inclusion parameters encompassed: (1) apparently healthy adults aged 18 to 60 years, with balanced gender representation; (2) Body Mass Index (BMI) within the normative range (18.5–23.9 kg/m^2^); (3) absence of documented food allergies or intolerances to test foods; (4) no use of any pharmacological agents affecting glucose metabolism (including hypoglycemic drugs, hormonal contraceptives, acetylsalicylic acid, corticosteroids, protease inhibitors and antipsychotic) in the 3 months prior to enrollment; (5) capacity to maintain a fasting period of at least 10 h prior to testing; (6) provision of signed informed consent.

The exclusion criteria encompassed: (1) a documented history of diabetes mellitus, impaired glucose tolerance, other metabolic syndromes, gastrointestinal diseases, endocrine disorders, or mental health conditions; (2) hypersensitivity to cereal proteins; (3) age under 18 or over 60 years; (4) irregular circadian behaviors, including chronic nocturnal activity or habitual late-night alimentation.

**Figure 1:**
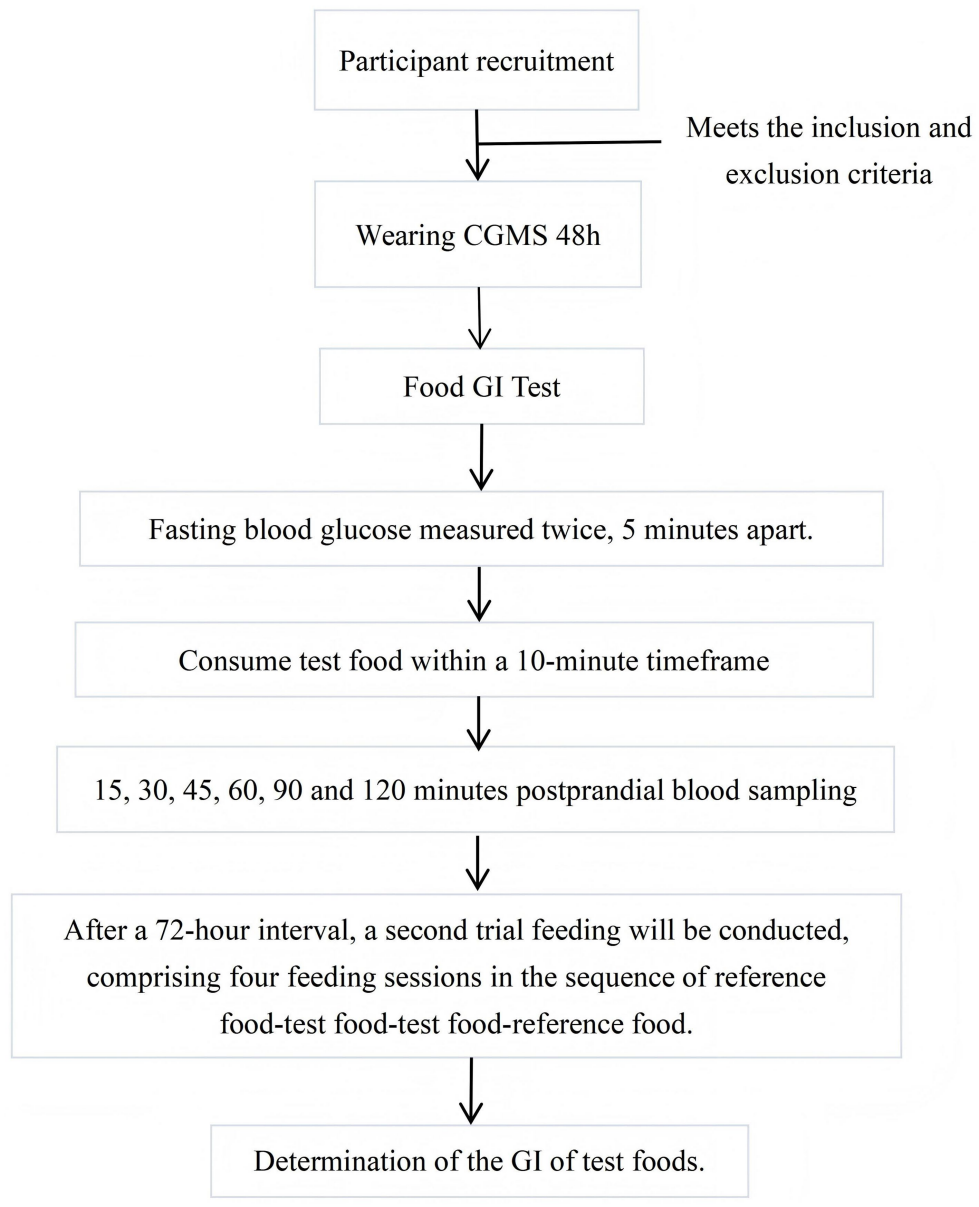
Flow chart of GI determination procedure in accordance with WS/T 652–2019.

According to WS/T 652–2019, the test sequence was strictly implemented in accordance with the following order: reference food test → test food test → test food test→ reference food test (the test food was arranged between two reference food tests to mitigate interference from residual effects). A clear flow chart was included to visually present the study design (Figure 1), detailing the entire process from participant screening, test sequence arrangement, to sample collection. Each test session was conducted with a minimum interval of 72 h between consecutive trials (serving as the washout period to eliminate residual effects of the previous test) (Figure 1). Participants were instructed to maintain their usual daily routines and consistent baseline dietary intake for 3 days before each test. The evening before testing, participants were advised to exclude high-fiber and high-sugar foods from their dinner and to abstain from eating after 10:00 p.m. On the testing day, strenuous physical activity was discouraged in the early morning, and participants were required to rest in a seated position for 10 min prior to sensory assessment. Two fasting venous blood samples were collected at 5-min intervals before the start of each test. Participants were instructed to consume the test substances and water within 5 to 10 min, with the timing starting at the first bite; the consumption duration was strictly controlled. Blood samples for glucose analysis were collected at 15, 30, 45, 60, 90, and 120 min post-ingestion, using venous blood from the antecubital vein. Venous blood was collected in gel serum separator tubes or vacuum tubes containing potassium oxalate-sodium fluoride anticoagulant. Following standard clinical laboratory procedures, the glucose concentration in each blood sample was measured at each time point using the hexokinase method.

The study protocol received approval from Medical Ethics Committee of Lianshui People’s Hospital Affiliated to Kangda College, Nanjing Medical University (Approval No: 20251126–05). This trial was also registered with the Chinese Clinical Trial Registry (registration number ChiCTR2500115033). All participants provided their written informed consent prior to study participation.

### Sample size setting and rationale

In according with the requirements of WS/T 652–2019, the number of subjects was determined to be a minimum of 12 participants.

### Continuous glucose monitoring system

The CGMS utilized in this study was the Yuwell Anytime 5H Pro (manufactured by Jiangsu Yuwell-Poct Biological Technology Co., Ltd. China, Medical Device Registration Certificate Number 20253070727 in China) (17). Regarding performance accuracy, this model exhibits a mean absolute relative difference (MARD) of 8.58% (18). Glucose data were automatically sampled every 3 min, yielding 480 data points per day. The device features factory-calibrated biosensor technology with the option for manual fingertip blood glucose calibration if required. Participants wore the device for 48 h preceding the formal trial to allow for a stabilization and calibration period.

### Test food

The test food, a dietary fiber-rich product manufactured by Nanjing Runjian Biotechnology Co., Ltd., comprised a macronutrient composition of 1,569 kcal energy, 30.5 g protein, 30.5 g fat, 42.6 g total carbohydrates, and 13.0 g dietary fiber per 100 g, with 15 mg sodium. The product’s manufacturing license is registered as SC10632012400228. A portion of 117.37 g was consumed in the study, delivering approximately 50 g of carbohydrates.

#### Reference food

In accordance with the universally accepted standard (16), 50 g of anhydrous glucose was used as the reference substance for dietary carbohydrate intake in this study.

### GI for test food

The GI of test food was determined following the WS/T 652–2019 protocol. Blood glucose response curves were generated by plotting time in minutes on the x-axis against blood glucose concentrations (mmol/L) on the y-axis for each subject after ingesting the various test foods. The incremental area under the curve (IAUC, mmol·min/L) was computed using geometric integration methods. The GI was calculated by comparing the IAUC values of the test food with those of two reference foods, averaging these ratios, and applying formulas (1) and (2) with the assistance of specialized analytical software.

$${\mathrm{GI}}_{n}=\text{IAUC of test food}/\text{IAUC of reference food}\times 100$$ (1)

$$\mathrm{GI}=\frac{\text{ΣGIn}}{n}$$ (2)

### Glucose measurement methods and comparative design

We compared three distinct methods for calculating the 120-min IACU and GI of the test food. The methodological rationale for employing three approaches (rather than solely contrasting venous sampling with a single CGMS-based method) was to comprehensively evaluate the applicability and value of CGMS in GI determination. The three methods are detailed as follows:

1. Method A (Venous Sampling, Gold Standard): Venous blood samples were collected at designated time points (0, 15, 30, 45, 60, 90, and 120 min post-ingestion) to measure glucose concentration. This served as the gold standard for evaluating the accuracy of CGMS-based methods.
2. Method B (CGMS with All Data Points Adopted): CGMS was performed throughout the 120-min test period, with all recorded CGMS data points were utilized to calculate IACU and GI. This method was designed to capture the continuous glucose response curve, providing high-resolution, real-time information on postprandial glucose fluctuations that may be missed by discrete venous sampling time points.
3. Method C (CGMS data aligned with the standard venous time points): CGMS data were extracted exclusively at the seven designated time points corresponding to venous sampling (0, 15, 30, 45, 60, 90, and 120 min post-ingestion). This method was specifically designed to enable direct comparability between CGMS and venous glucose measurements at the standard time points specified by clinical guidelines, thereby verifying the consistency of CGMS with the gold-standard venous sampling at clinically relevant time points.

A comparative analysis of the results obtained from the three methods, along with an evaluation of their methodological concordance, was conducted to assess the performance of CGMS-based methods relative to the gold-standard venous sampling.

### Statistical analysis

All statistical evaluations were performed utilizing IBM SPSS Statistics version 26.0. Parametric data with normal distribution were summarized as mean ± standard deviation, whereas non-parametric data were presented as median with interquartile range. The IAUC was used as the primary outcome measure for subsequent statistical analysis. One-way analysis of variance (ANOVA) followed by the LSD t -test was employed to compare the differences in IAUC and GI values among the three methods. Methodological concordance was assessed using Lin’s Concordance Correlation Coefficient (CCC). A two-tailed *p* value < 0.05 was considered statistical significant. Parkes consensus error grid analysis was applied to assess the clinical agreement between paired reference and tested blood glucose readings. Measurements were classified into five distinct clinical risk zones in accordance with established Parkes error grid thresholds. Scatter diagrams were constructed, including the line of perfect equality and standardized zone boundaries. The percentage distribution of data points across Zone A - E was quantified, and clinical risk stratification was conducted following ISO 15197 specifications. All statistical and graphical procedures were implemented in base R.

## Results

### Baseline characteristics of the participants

This study involved 16 participants. Following the exclusion of individuals who did not meet BMI criteria and presented abnormal oral glucose tolerance test (OGTT) results, 12 participants were retained (six male and six female) for statistical analysis. The demographic and biochemical parameters for these 12 participants were summarized in Table 1. The cohort had a mean age of 29.50 ± 10.12 years and a mean BMI of 21.5 ± 1.7 kg/m^2^. The mean glycated hemoglobin (HbA1c) was 5.07 ± 0.21%, and mean hemoglobin was 145.50 ± 19.36 g/L. All measured indices of lipid profile, glycemia, and hepatic and renal function were within standard reference ranges.

**Table 1:** Patients’ characteristics.

Characteristics (mean ± SD)	Total = 12
Age, year	30.08 ± 9.49
Male, n (%)	6 (50%)
BMI, kg/m^2^, mean ± SD	21.5 ± 1.7
Glycated hemoglobin A1c (HbA1c), %	5.07 ± 0.21
Hemoglobin (Hb), g/L	144.92 ± 17.98
Total-cholesterol (TC), mmol/L	4.55 ± 0.74
Triglyceride (TG), mmol/L	1.08 ± 0.55
High-density lipoprotein cholesterol (HDL-C), mmol/L	1.35 ± 0.28
Low-density lipoprotein cholesterol (LDL-C), mmol/L	2.47 ± 0.37
Lipoprotein (a) [Lp(a)], mg/L	130.53 ± 77.20
Glucose (GLU), mmol/L	5.10 ± 0.97
Urea, Creatinine, μmol/L	62.42 ± 16.23
Uric Acid, μmol/L	300.92 ± 82.89
Alanine aminotransferase (ALT), U/L	14.92 ± 6.33
Aspartate aminotransferase (AST), U/L	17.67 ± 4.50
γ-Glutamyl transferase (*γ*-GGT), U/L	19.92 ± 10.01

### Parkes error grid analysis

**Figure 2:**
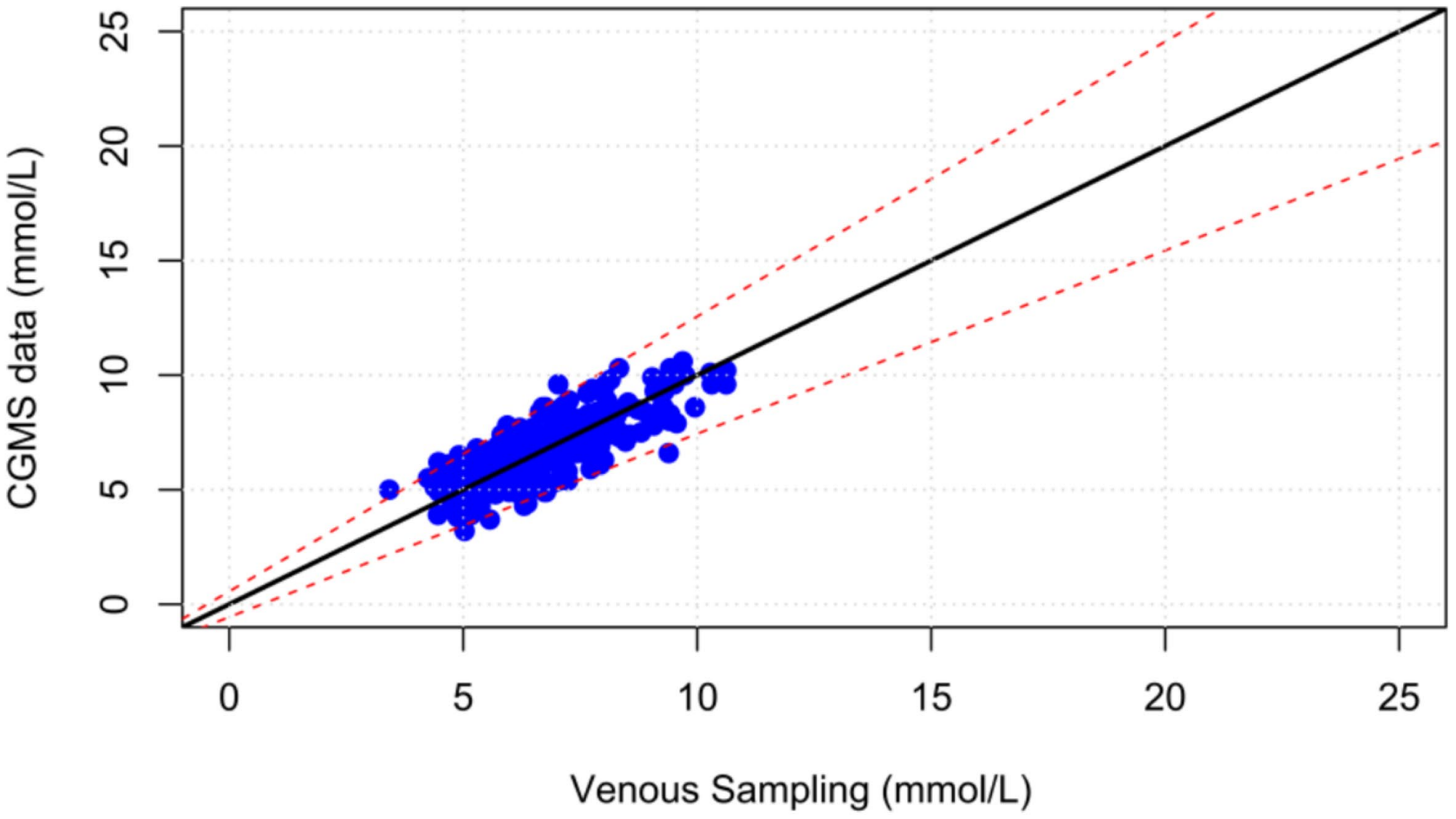
Parkes consensus error grid comparing venous sampling and continuous glucose monitoring system (CGMS) measurements.

All paired venous reference glucose and CGMS measurements were included in the Parkes consensus error grid analysis (Figure 2). 97.396% of all data points fell within Zone A, with 2.344 and 0.260% in Zone B and C, respectively. No clinically dangerous deviations were identified across the full glycemic range. The glucose monitoring system fully met the ISO 15197 requirements for clinical accuracy. All measurements showed excellent consistency along the ideal y = x concordance line, with tight aggregation and no obvious outlier deviation.

### Descriptive statistics and inter-method comparison of IAUC values

All three methods yielded n = 48 IAUC measurements. Mean 120-min IAUC values were 186.93 ± 87.29 for Method A, 166.00 ± 76.46 for Method B, and 164.24 ± 77.48 for Method C (Table 2). Levene’s test for homogeneity of variance indicated no significant difference in variance across groups (*p* > 0.05). ANOVA revealed no statistically significant difference in mean IAUC values between methods (*F* = 1.178, *p* = 0.311). Post-hoc analyses (LSD tests) confirmed no significant pairwise differences (all *p* > 0.05). Detailed results were presented in Tables 3, 4.

**Table 2:** The mean IAUC of three methods.

Method	n	mean	SD	SE	95% CI	
					Lowe	Upper
Method A	48	186.9294	87.28538	12.59856	161.5843	212.2744
Method B	48	165.9952	76.46254	11.03642	143.7928	188.1976
Method C	48	164.2427	77.48260	11.18365	141.7441	186.7413
Total	144	172.3891	80.65857	6.72155	159.1027	185.6755

**Table 3:** Analysis of variance for IAUC from different methods.

Source of variation	Sum of squares	Degrees of freedom	Mean square	*F*	*P*
Inter-group	15,295.927	2	7,647.963	1.178	0.311
Intra-group	915,034.066	141	6,489.603		
Total	930,329.993	143			

**Table 4:** Multiple comparisons for IAUC from different methods.

Test	Factor (I)	Factor (J)	Difference between means (I-J)	Standard error	*P*	95% CI	
						Lower	Upper
LSD	Method A	Method B	20.93417	16.44385	0.205	−11.5742	53.4425
		Method C	22.68667	16.44385	0.170	−9.8217	55.1950
	Method B	Method A	−20.93417	16.44385	0.205	−53.4425	11.5742
		Method C	1.75250	16.44385	0.915	−30.7559	34.2609
	Method C	Method A	−22.68667	16.44385	0.170	−55.1950	9.8217
		Method B	−1.75250	16.44385	0.915	−34.2609	30.7559

### Concordance analysis of IAUC values

Using the IAUC from Method A as the reference standard, we performed concordance analyses with the IAUC from other two CGMS-based methods (Table 5). The CCC between Method A and Method B was 0.8818 (95% CI: 0.8068–0.9289), with a Pearson’s *ρ* of 0.9191 and a bias correction factor (Cb) of 0.9594, indicating good agreement with minimal systematic bias. For method A and method C, the CCC was 0.8727 (95% CI: 0.7921–0.9234), ρ was 0.9129, and Cb was 0.9561, also demonstrating good agreement. Collectively, these results indicated strong agreement and interchangeability between Method A and Methods B/C.

**Table 5:** Concordance analyses among IAUC from three methods.

Item	CCC	95%CI	Pearson ρ	bias correction factor (Cb)
Method A and B	0.8818	0.8068–0.9289	0.9191	0.9594
Method A and C	0.8727	0.7921–0.9234	0.9129	0.9561

### Descriptive statistics and inter-method comparison of GI values

Each method produced n = 12 GI values. The mean GI was 55.07 ± 15.57 for Method A, 54.28 ± 12.24 for Method B, and 53.68 ± 15.04 for Method C, with a pooled mean of 54.34 ± 13.95. Variance was homogeneous across groups (Levene’s test, *p* > 0.05). ANOVA revealed no significant inter-method difference in mean GI (*F* = 0.028, *p* = 0.972). Post-hoc comparisons confirmed no significant pairwise differences (all p > 0.05). Results were presented in Tables 6, 7.

**Table 6:** The mean GI of three methods.

Method	*n*	Mean	SD	SE	95% CI	
					Lowe	Upper
Method A	12	55.0650	15.56996	4.49466	45.1723	64.9577
Method B	12	54.2812	12.24319	3.53430	46.5023	62.0602
Method C	12	53.6801	15.03636	4.34062	44.1265	63.2338
Total	36	54.3421	13.95308	2.32551	49.6211	59.0632

**Table 7:** Analysis of variance for GI from different methods.

Source of variation	Sum of squares	Degrees of freedom	Mean square	*F*	*P*
Inter-group	11.575	2	5.787	0.028	0.972
Intra-group	6802.524	33	206.137		
Total	6814.098	35			

### Concordance analysis of GI values

Using GI from Method A as the reference, concordance with Method B/C was assessed (Tables 8, 9). The CCC for Method A versus Method B was 0.7898 (95% CI: 0.4616–0.9278), with *ρ* of 0.8141 and Cb of 0.9701, indicating moderate agreement without apparent systematic bias. For Method B versus Method C, the CCC was 0.7954 (95% CI: 0.4378–0.9357), ρ was 0.7995, and Cb was 0.9949, also showing moderate agreement with minimal systematic bias.

**Table 8:** Multiple comparisons for GI from different methods.

Test	Factor (I)	Factor (J)	Difference between means (I-J)	Standard error	*P*	95% CI	
						Lower	Upper
LSD	Method A	Method B	0.78377	5.86141	0.894	−11.1414	12.7089
		Method C	1.38491	5.86141	0.815	−10.5402	13.3100
	Method B	Method A	−0.78377	5.86141	0.894	−12.7089	11.1414
		Method C	0.60114	5.86141	0.919	−11.3240	12.5263
	Method C	Method A	−1.38491	5.86141	0.815	−13.3100	10.5402
		Method B	−0.60114	5.86141	0.919	−12.5263	11.3240

**Table 9:** Concordance analyses among the GI from three methods.

Item	CCC	95%CI	Pearson ρ	Bias correction factor (Cb)
Method A and B	0.7898	0.4616–0.9278	0.8141	0.9701
Method A and C	0.7954	0.4378–0.9357	0.7995	0.9949

## Discussion

This study assessed baseline characteristics of healthy participates and compared IAUC and GI values obtained from venous blood sampling with those derived from two CGMS approaches. This study strictly adhered to China’s industry standards for determining the glycemic index of foods, particularly Method A. The results indicated good-to-moderate agreement and interchangeability among the three methods, supporting the reliability of CGMS-derived metrics for postprandial glycemic assessment in healthy, normal-weight, normoglycemic participants. The implementation of this study underscores the following key aspects.

Firstly, although CGMS has been widely used to assess glycemic control, glucose variability, and treatment response in patients with diabetes (14, 19), most studies have not directly investigated their correlation with the glycemic index of foods. A randomized crossover trial (NCT06333184) compared the accuracy of CGM versus traditional capillary blood sampling in determining the GI of food among 15 healthy adult subjects (20). The findings revealed that CGM systematically overestimated the glycemic response, resulting in a calculated GI value significantly higher than that obtained via capillary blood sampling (CGM: 69 vs. Capillary: 53, *p* = 0.05). Hutchins’s study (20) suggests that while CGM provides valuable, continuous dynamic glucose data, its use in deriving food GI values may be subject to bias. Khalil et al. (21) examined glycemic response to meals containing dates using the Minimed-530 g-Enlite CGMS and glucometer in 20 patients with type 2 diabetes and 20 healthy controls. The results demonstrated no significant differences in the GI between various date-based meals. Bakhshi et al. (22) employed only CGM to investigate the impact of carbohydrate quality on blood glucose in 677 non-diabetic individuals from the Framingham Heart Study. The results revealed that consumption of high-fiber carbohydrates—such as those containing at least 1 gram of fiber per 9 grams of carbohydrate—was associated with reduced time spent above a blood glucose level of 140 mg/dL. Interestingly, the blood glucose values used as reference controls in the Hutchins’s and Khalil’s studies all originated from glucometers rather than the gold standard venous blood glucose measurements. In conclusion, the application of CGMS in determining the GI of foods remains in the exploratory phase, with limited related research and inconsistent findings. In contrast to previous research, this study is the first to determine the GI values of foods based on China’s national standardized GI assessment method. Furthermore, it provides a direct comparison between the GI values determined by this standard method and those assessed using CGMS.

Secondly, one way ANOVA revealed that there was no significant inter-method differences among GI values (*F* = 0.028, *p* = 0.972). Mean GI values were nearly identical across methods. Unlike previous studies, this research not only employed analysis of variance to compare GI values but also utilized Lin’s Concordance Correlation Coefficient (CCC) together with Person *ρ* and bias correction factors (Cb) to conduct a consistency analysis across the different methods. Lin’s Concordance Correlation Coefficient, known as the CCC, is a statistical measure used to assess the “degree of exact agreement” between two variables and has been widely applied across numerous fields including medicine, nutrition, and environmental science (23). Notably, Lin’s CCC has been employed across multiple biomedical testing platforms to evaluate the consistency between laboratories, devices, or methodologies (24). The CCC not only measures the linear association between two variables but also simultaneously assesses their agreement in means and similarity in variances. This makes it particularly suitable for evaluating concordance—rather than merely correlation—between two measurement methods or repeated measurements (25). The Pearson *ρ* measures the strength and direction of linear relationships between two continuous variables, with values ranging from −1 to 1. It solely reflects association, rather than agreement or systematic bias (26). Cb is used to adjust for systematic bias caused by measurement error, sampling design, or the statistical methodology itself, thereby achieving more accurate effect estimates (27). These three methods are often used in combination. For instance, Pearson’s ρ is first employed to detect strong relationships. CCC is then applied to assess potential interchangeability.

In this study, the observed agreement levels support CGMS as a valid alternative to venous sampling for IAUC and GI measurement in healthy populations. In this study, concordance analysis yielded moderate agreement (CCC ≈ 0.79), consistent with acceptable reproducibility for GI determination. While slightly lower than IAUC agreement, these CCC values meet clinical acceptance thresholds for nutritional epidemiology and food glycemic ranking (28, 29). The use of Lin’s CCC improved the comparability and reproducibility of the GI data. Unlike Pearson correlation alone, CCC quantifies deviation from perfect equality, making it particularly suitable for validating glucose monitoring systems (11, 12). Under the conditions of this study, the comparable precision and accuracy observed across all three methods support the potential use of CGM as a feasible tool for rapid, high-throughput GI testing in healthy, normal-weight, normoglycemic individuals. However, given the moderate agreement for GI values, small sample size, and single test food employed, these findings should be interpreted with caution, and further research in larger and more diverse populations is warranted to confirm its generalizability.

Thirdly, unlike prior studies which solely performed variance analysis on glucometer and CGM-derived GI values, this research also conducted variance analysis and consistency tests on the IAUC derived from venous blood and continuous glucose monitoring systems. Similar to the GI comparison, this study employed both analysis of variance and Lin’s concordance correlation coefficient for the IAUC comparison. One-way ANOVA showed no significant inter-method differences (*F* = 1.178, *p* = 0.311), with homogeneous variance across groups among IAUC values obtained by different methods. Lin’s concordance analysis using venous IAUC as the reference revealed good agreement for both CGMS methods: CCC = 0.8818 (A vs. B) and 0.8727 (A vs. C), accompanied by high Pearson correlation (>0.91) and bias correction factors (>0.95). These findings align with previous reports showing strong correlation between CGMS and venous glucose in euglycemic individuals (30). The high Cb values further confirm minimal systematic bias, indicating that CGMS-derived IAUC reliably reflects total postprandial glycemic exposure (31).

One way ANOVA for GI and IAUC, along with CCC, demonstrates that CGMS may serve as a potential alternative to the conventional venous blood glucose measurement method for assessing the GI of foods in healthy, normal-weight, normoglycemic participants. In addition, the blood glucose profiles obtained by CGMS appear to show generally acceptable consistency with those from traditional venous blood glucose measurements at corresponding time points.

Fourthly, several notable patterns emerged from our analysis. First, among volunteers with HbA1c levels above 5%, Methods B and C showed significantly higher CCC value with Method A for determining GI values compared to the normoglycemic population. Second, GI values obtained from Methods B and C had higher CCC value compared with those from Method A in female participants than in male participants. Third, in volunteers aged 30 and above, GI values derived from Methods B and C demonstrated considerably higher CCC value compared with those from Method A. These phenomena are the focal point of our research group’s subsequent in-depth investigation.

In summary, venous and CGMS-based methods yielded similar IAUC and GI values with fair to moderate concordance. CGMS enables continuous, non-invasive monitoring with reasonable accuracy suggesting its potential utility in postprandial glycemia research and food GI assessment. This study was consisted of normoglycemic, non-obese young adults with normal lipid, hepatic, and renal function, providing a relatively stable baseline for method comparison with limited confounding from metabolic disorders. Strict exclusion criteria supported participant homogeneity and may have reduced physiological variability that could influence glycemic responses (32).

Of course, several limitations should be acknowledged. First, the small sample size (*n* = 12) and homogeneous normoglycemic, healthy participants reduce result generalizability and statistical power. Second, CGMS performance in insulin-resistant, obese, or diabetic populations remains untested, limiting clinical relevance. Third, only one CGMS brand/model was used, and insufficient device details hinder reproducibility. Fourth, a single test food with incomplete descriptions affects result interpretation and reproducibility. Future studies with larger, diverse cohorts, multiple CGMS devices, detailed test food/design descriptions, and clinical risk assessment are warranted; machine learning-augmented CGM may offer new directions (33).

## Conclusion

This study innovatively assessed the consistency of three methods for measuring GI in accordance with China’s industry standards (WS/T 652–2019). The analysis used both venous blood glucose measurement (the gold standard for GI determination) and CGMS approaches, following standardized protocols to ensure methodological rigor. GI was estimated by monitoring participants’ postprandial blood glucose responses via CGM devices, which can provide continuous, detailed data to assist in calculating the IAUC. This approach may allow for alignment with the traditional venous sampling method by evaluating glucose levels at comparable time points, though its generalizability to broader populations and different test foods requires further verification.

## Data Availability

The raw data supporting the conclusions of this article will be made available by the authors, without undue reservation.
